# Research on the soothing Liver — Qi stagnation method in the treatment of postoperative papillary thyroid carcinoma patients’ concomitant depression: A randomized controlled clinical trial

**DOI:** 10.1097/MD.0000000000039325

**Published:** 2024-09-13

**Authors:** Huiyue Lin, Xueting Zhang, Yuqian Zheng, Chenchen Tang, Juyong Wang

**Affiliations:** a Oncology Department, Longhua Hospital Shanghai University of Traditional Chinese Medicine, Shanghai, China; b Department of Oncology and Hematology, Wenzhou Hospital of Integrated Traditional Chinese and Western Medicine, Wenzhou, Zhejiang Province, China; c Longhua Hospital, Shanghai University of Traditional Chinese Medicine, Shanghai, China; d Department of Experimental Management, School of Integrative Medicine, Shanghai University of Traditional Chinese Medicine, Shanghai, China.

**Keywords:** 5-hydroxytryptamine, Hamilton depression scale: randomized controlled clinical trial, postoperative papillary thyroid cancer with depression, soothing Liver-Qi stagnation method

## Abstract

**Background:**

Postoperative papillary thyroid carcinoma (P-PTC) patients often grapple with depression fueled by the looming threat of recurrence. While the Liver-Qi stagnation method is frequently employed for depression management, a notable scarcity of clinical trials exists regarding its application in patients with P-PTC and concurrent depression. This study presents a randomized controlled clinical trial, aiming to establish the efficacy of the Liver-Qi stagnation method in alleviating depression in patients with P-PTC.

**Methods:**

In this randomized controlled clinical trial, P-PTC patients diagnosed with concomitant depression were systematically enrolled. Subjects were randomly assigned to either the control or test group, both receiving standard treatment comprising Levothyroxine sodium tablets and decoction of benefiting Qi and nourishing Yin. Additionally, the test group received supplementation with bupleuri radix-paeoniae alba radix (CH-BS) alongside the baseline therapy. The intervention spanned 12 weeks. Pre- and post-treatment evaluations were conducted using the Hamilton Depression Scale (HAMD), European Organization for Research and Treatment of Cancer Quality of Life Questionnaire (EORTC QLQ-C30) and Traditional Chinese Medicine (TCM) syndrome score scale. Concurrently, blood inflammatory factors and serum 5-hydroxytryptamine (5-HT) levels were measured to comprehensively assess treatment outcomes.

**Results:**

During the 12-week intervention, the test group demonstrated a significant reduction in HAMD scores compared to the control group (*P* < .05). Moreover, post-treatment serum 5-HT levels were significantly elevated in the test group compared to the control group (*P* < .05). Findings gleaned from the EORTC QLQ – C30 revealed a noteworthy improvement in social function and overall quality of life scores within both groups post-treatment in comparison to baseline (*P* < .05). Concurrently, post-treatment scores for fatigue and insomnia symptoms witnessed a significant decrease compared to baseline (*P* < .05). Notably, the test group exhibited superior scores in the emotional domain in contrast to the control group (*P* < .05). Both groups exhibited a substantial decrease in TCM syndrome scores from baseline (*P* < .05). Noteworthy increases were found in IFN-γ < 2.44 rate (62.86%) and IL-6 < 2.44 rate (74.29%) in the test group compared to pretreatment levels (*P* < .05).

**Conclusion:**

The soothing Liver-Qi stagnation method induces a rise in serum 5-HT levels, reducing depression-related inflammatory factors, culminating in the alleviation of depression for P-PTC.

## 1. Introduction

Thyroid cancer stands as one of the prevalent malignancies, with global new cases estimated at around 580,000 in 2020, as per incomplete statistics from the World Health Organization (WHO) (https://gco.iarc.fr/). Approximately 59.7% of these cases are reported in Asians. According to the International Agency for Research on Cancer, the number of new thyroid cancer cases in China is 190,000 in 2022.^[1]^ These statistics underscore the substantial patient population affected by thyroid cancer in China. The pathological type of thyroid cancer includes Papillary Thyroid Carcinoma (PTC), Follicular Thyroid Carcinoma, Medullary Thyroid Carcinoma, Anaplastic Thyroid Cancer, and Undifferentiated Thyroid Carcinoma. PTC, constituting 85% to 90% of all thyroid cancers, emerges as the most prevalent in clinical practice.^[2]^ Research underscores the intrinsic connection between psychological factors and the onset of malignant tumors, with thyroid cancer being no exception.^[3]^ Despite the improved prognosis following surgery, Postoperative papillary thyroid carcinoma(P-PTC) patients bear a psychological burden akin to or surpassing that of patients with other malignancies.^[4,5]^ A study reported recurrence rates of PTC at 10, 20, and 30 years post-diagnosis as 11.3%, 21.8%, and 29.4%, respectively.^[6]^ Importantly, the fear of recurrence imposes a distinct psychological burden on these patients, predominantly presenting as depression.^[7]^ Depression, with its documented carcinogenic effects, influences the release of stress hormones implicated in relevant signaling pathways, regulates cell growth and pericellular sufficients, as well as inducing impairment of immune function and affecting immune surveillance.^[8]^ Moreover, the presence of depression also can significantly impact treatment adherence among P-PTC patients, thereby influencing their prognosis and quality of life.^[9,10]^

PTC is currently managed through a comprehensive three-step treatment regimen comprising surgery, Iodine-131 (^131^I) therapy, and thyroid hormone suppression.^[9]^ Thyroid stimulating hormone (TSH) suppression therapy constitutes a pivotal aspect of post-thyroidectomy long-term management. By administering thyroxine over an extended period to maintain TSH levels at or below the lower limit of the normal range, this regimen not only mitigates the risk of thyroid hormone deficiency but also effectively suppresses the recurrence or metastasis of thyroid cancer.^[11,12]^ The intricate relationship between thyroid hormones and depression has been well-established.^[13]^ Prolonged reliance on thyroxine supplementation post-thyroidectomy may predispose individuals to varying degrees of subclinical hyperthyroidism, thus heightening susceptibility to depressive states among post-thyroid cancer patients.^[14]^ Depression, a pervasive affliction, assumes particular significance within the oncological context.^[15]^ Serum concentrations of 5-hydroxytryptamine (5-HT) emerge as pivotal biomarkers, exhibiting strong associations with both depression and thyroid disorders. Furthermore, they have been implicated in tumorigenesis and disease progression.^[16–18]^ Within depressed cohorts, diminished levels of 5-HT coincide with elevated kynurenine, a metabolite implicated in immune evasion by tumor cells.^[19,20]^

The connection between emotional factors and thyroid cancer development has been elucidated in Chinese medicine.^[21]^ Notably, patients diagnosed with thyroid cancer often grapple with concurrent depression.^[7]^ When managing thyroid cancer patients with coexisting depression, the integration of antidepressant therapy alongside antitumor interventions becomes imperative. Traditional Chinese medicine (TCM), an intrinsic medical paradigm in China, enjoys widespread recognition as a crucial complementary and alternative medicine with discernible benefits for cancer patients.^[15]^ The TCM theory identifies “Liver qi stagnation” as a primary cause of depression, making the soothing Liver-Qi stagnation a key therapeutic avenue.^[22]^ Chinese Herbal Medicine (CHM), a facet of TCM, not only demonstrates anti-cancer properties but also exerts antidepressant effects. These effects are orchestrated through a myriad of chemical components, acting on diverse targets and engaging multiple pharmacological mechanisms.^[23,24]^ Radix Bupleuri-Radix Paeoniae Alba (CH-BS, known as chaihu-baishao in Chinese) constitutes a classical herb pair widely employed in clinical settings to address depression by soothing Liver-Qi stagnation. Beyond its antidepressant properties, CH-BS demonstrates efficacy in combating tumors.^[25]^ To investigate the clinical efficacy of the soothing Liver-Qi stagnation method in alleviating depression among P-PTC patients with concomitant Qi-Yin deficiency, this study undertakes a randomized controlled trial. The objective is to furnish a robust scientific foundation for the clinical integration of the soothing Liver-Qi stagnation method in treating P-PTC patients grappling with depression.

## 2. Methods

### 2.1. Study population

**Figure 1.**
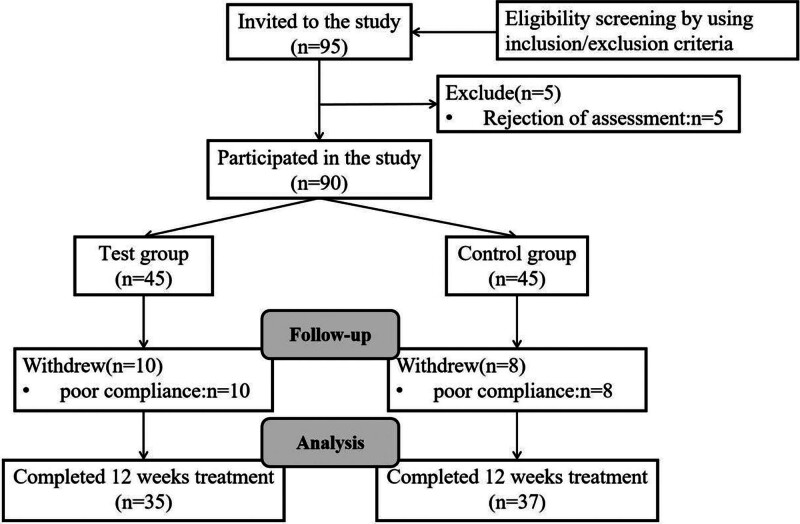
Flowchart of the study recruitment.

A cohort comprising 72 P-PTC patients with Qi-Yin deficiency, characterized by depression, was meticulously recruited for this randomized controlled clinical trial. The study transpired at Longhua Hospital, Shanghai University of Traditional Chinese Medicine, spanning from August 2020 to February 2022 (Fig. 1). Adhering to the ethical standards outlined in the Declaration of Helsinki, the study received approval from the Ethics Committee of Longhua Hospital, Shanghai University of Traditional Chinese Medicine, Shanghai, PRC (Approval Number: 2020LCSY057). Thorough information regarding the study’s objectives and procedures was imparted to all outpatients, who, in turn, provided informed consent before participation.

### 2.2. Diagnostic criteria

#### 2.2.1. Diagnostic criteria of western medicine

The diagnosis of thyroid cancer adhered to the guidelines outlined in the Thyroid Cancer Diagnostic and Treatment Guidelines (2018 Edition) in China^[26]^ and the 2015 American Thyroid Association Management Guidelines for adult patients with thyroid nodules and differentiated thyroid cancer.^[12]^ Specifically, patients whose postoperative pathology confirmed the diagnosis of PTC were included. To identify cancer-related depression, we followed the criteria set forth in the International Classification of Diseases 11th revision (ICD-11).^[27]^ Depressive symptoms were further assessed using the Hamilton Depression Scale-24 Items (HAMD-24), with a score exceeding 8 indicating the presence of depression.^[28]^

#### 2.2.2. Diagnostic criteria of TCM

To discern Qi-Yin deficiency within Traditional Chinese Medicine (TCM) syndromes, we referenced the findings presented in “TCM patterns types and indicators of thyroid cancer by Delphi method: an investigation,”^[21]^ coupled with guidance outlined in the “Guiding Principles for Traditional Chinese Medicine and New Medicine on Clinical Trials.”^[29]^

### 2.3. Selection criteria

Inclusion criteria encompassed: individuals aged 18 to 65 years; patients meeting the Western medical diagnostic criteria for pathology, specifically PTC; patients meeting the TCM syndromes diagnostic criteria for Qi-Yin deficiency; the duration of P-PTC within 3 years; adherence to levothyroxine sodium tablets as foundational treatment post-surgery for PTC; absence of TCM treatment within the past 2 weeks; HAMD-24 score falling within the range of 8 to 35; and Karnofsky Performance Status (KPS) ≥ 80.

Exclusion criteria comprised: patients necessitating other medications due to a change in condition; recent exposure (within 2 weeks) to radiotherapy, chemotherapy, immunotherapy, or targeted therapy; concomitant presentation of other tumors, bipolar disorder, schizophrenia, or infectious diseases; and coexistence of severe primary diseases affecting the hematopoietic system, heart, brain, kidney, liver, respiratory system, or other critical organ systems. Patients meeting any of the above criteria were excluded from this clinical trial.

Drop-out criteria were defined as: voluntary withdrawal from the trial by the patients themselves; and poor compliance leading to an impairment in the evaluation of efficacy and safety.

### 2.4. Randomization and intervention

#### 2.4.1. Randomization

Utilizing the randomization scheme generated by IBM SPSS software (version 26.0, IBM Corp., NY), patients were allocated into either the test group or the control group, adhering to a 1:1 ratio. While this study did not implement blinding for subjects and physicians during the actual operation, a meticulous blinding approach was adopted for personnel involved in data collection, efficacy evaluation, and data statistics. These individuals remained uninformed about the specific treatment processes and group assignments, thereby upholding a segregation of operation, evaluation, and statistics to mitigate potential biases.

#### 2.4.2. Intervention

In addition to the study medication, the consumption of other herbal remedies and treatments related to antidepressants was strictly prohibited throughout the observation period. Adhering to the standard of thyrotropin suppression therapy outlined in the Thyroid Cancer Diagnostic and Treatment Guidelines (2018 Edition) in China,^[26]^ all enrolled patients maintained a stable dose of levothyroxine sodium tablets for a minimum of 3 months.

Test group: levothyroxine sodium tablets + decoction of benefiting qi and nourishing yin (Astragalus flavone 30 g, Atractylodes Rhizome 12 g, Pinellia ternata 12 g, Rhizoma Smilacis Glabrae 30 g, Ganoderma lucidum 30 g, Bulb of Thunberg Fritillary 12 g, Prince Ginseng 12 g, Rhizoma Arisaematis 12 g, cicada slough 12 g, Hive 9 g, Fructus Ligustri Lucidi 15 g) + CH-BS (Radix Bupleuri 12 g, Radix Paeoniae alba 12 g).

Control group: levothyroxine sodium tablets + decoction of benefiting qi and nourishing yin (Astragalus flavone 30 g, Atractylodes Rhizome 12 g, Pinellia ternata 12 g, Rhizoma Smilacis Glabrae 30 g, Ganoderma lucidum 30 g, Bulb of Thunberg Fritillary 12 g, Prince Ginseng 12 g, Rhizoma Arisaematis 12 g, cicada slough 12 g, Hive 9 g, Fructus Ligustri Lucidi 15 g).

The decoction of herbs were taken twice daily, consumed warm half an hour after meals. The treatment spanned a total of 12 weeks.

### 2.5. Sample size estimation

In the preliminary phase, the test group exhibited an antidepressant efficacy of 85%, while the control group demonstrated a 50% efficacy. Guided by these preexperimental findings and setting a two-sided α of 0.05 with a confidence level of 90%, we employed the two-proportions test in PASS software (version 15, NCSS, LLC, Kaysville, Utah) to calculate the sample size for the treatment and control groups. The resulting sample size for both groups was determined to be 33 cases each. Accounting for a potential 20% loss due to missed visits or refusals, a minimum of 42 study subjects per group was deemed necessary, summing up to a total of 84 cases.

### 2.6. Observation indicators

#### 2.6.1. Primary clinical outcome indicators

The depression status of all participants underwent evaluation utilizing HAMD-24,^[30]^ a widely utilized tool in Chinese clinical practice for assessing depression. The HAMD-24 encompasses 7 domains encompassing anxiety/somatization, weight, diurnal variation, cognitive disorder, retardation, sleep disorder, and hopelessness. Face-to-face interviews conducted by 2 trained psychiatrists were employed to assess the HAMD-24 scores. Patients registering HAMD scores above 8 were identified as exhibiting depressive symptoms, and a treatment response was defined as a 25% reduction in the HAMD-24 score from baseline. The overall effective rate denotes the proportion of treatment responses in the total number of cases.^[31]^

#### 2.6.2. Secondary clinical outcome indicators

Blood samples underwent immediate centrifugation (100× g for 20 minutes), and the resultant serum samples were promptly collected and stored at −80°C until subsequent analysis. Serum 5-HT levels were quantified using a human serum 5-HT ELISA kit (Shanghai Zhen Ke Biological Technology, Shanghai, China) following the manufacturer’s instructions.

The Chinese version of the European Organization for Research and Treatment of Cancer Quality of Life Questionnaire (EORTC QLQ-C30; version 3.0) was employed in this study.^[32,33]^ This questionnaire encompasses functional scales including physical, role, emotional, cognitive, and social functioning, alongside symptom sub-scales such as pain, fatigue, nausea and vomiting, and global health status. Individual measurement items span appetite, insomnia, dyspnea, constipation or diarrhea, and economic status. All patients completed the EORTC QLQ-C30 (Chinese version 3.0) questionnaire themselves, with illiterate patients or those facing writing difficulties receiving assistance from doctors. Standardized health-related quality of life (HRQoL) scores were computed on a scale of 0 to 100, where elevated scores denoted superior functional levels, heightened global quality of life, and more pronounced symptoms.^[34]^

Certified Chinese medicine physicians, designated as chief practitioners, completed the TCM syndrome diagnosis questionnaire for Qi-Yin deficiency in PTC based on the TCM status of PTC patients. The questionnaire, tailored for Qi-Yin deficiency in PTC, comprises 14 closed-ended items. Each item is assigned a ranked scale with succinct descriptions delineating the severity or frequency of signs or symptoms. These 14 items are categorized into dimensions encompassing palpable anterior neck lump, fatigue and weakness, weakness of speaking, among others. Refer to Table 1 for the comprehensive list of items. A treatment response was defined as a 30% reduction in the TCM questionnaire score from baseline, and the overall effective rate denotes the ratio of treatment responses to the total number of cases.^[29]^

**Table 1:** Comprehensive 14-item TCM syndrome diagnosis questionnaire for Qi-Yin deficiency in PTC.

1. Palpable anterior neck lump
2. Fatigue and weakness
3. Weakness of speaking
4. Subjective perception of heat in 5 centers
5. Depressed spirit
6. Hoarseness of voice
7. Emaciation
8. Diurnal and nocturnal sweating
9. Vertigo and tinnitus
10. Tachycardia
11. Xerostomia and pharyngeal dryness
12. Few or pale yellow moss on the tongue
13. Heavy, tender, and rapid pulse
14. Pale red or red of tongue color

PTC = papillary thyroid carcinoma, TCM = traditional Chinese medicine.

Blood inflammatory factors (IFN-γ, IL-1β, IL-8, tumor necrosis factor α [TNF-α], IL-6) and thyroid function markers (TSH, T4, T3, Tg, TgAb) were measured using the hospital’s standardized instruments. Fasting blood draws were conducted both before and after treatment for subjects participating in the clinical study. In accordance with the Thyroid Cancer Diagnostic and Treatment Guidelines (2018 Edition) in China,^[26]^ TSH levels in thyroid function achieved the target for TSH suppression therapy, and the remaining indexes fell within the appropriate range, indicative of a favorable response.

### 2.7. Statistical analysis

Data analysis employed SPSS 26.0 statistical software (SPSS). For count data, between-group comparisons utilized the χ^2^ test, inclusive of the CMH-χ^2^ test, or Fisher’s exact probability method. Paired count data were assessed using the McNemar formula test. Measurement information was presented as mean ± SD (x ± s). The independent samples *t* test was applied for normally distributed data in group designs, while the Mann–Whitney *U* test was employed for skewed distributions. Paired design analyses of normally distributed data used the paired *t* test, whereas skewed distribution was assessed through the Wilcoxon signed-rank sum test. Statistical significance was established at *P* < .05.

## 3. Results

### 3.1. Participant characteristics

A total of 72 patients successfully completed this clinical trial. The final data analysis encompassed 35 patients in the test group and 37 patients in the control group. The general baseline characteristics of the enrolled patients, including age, gender, height, weight, medical insurance, occupation, education, marital status, fertility status, HAMD scores stratification, and various attributes pertinent to PTC such as gene mutation, lymphatic metastasis, cancer stage, I131 treatment, dose of Levothyroxine sodium tablets were meticulously compared. The results revealed no statistically significant difference in the baseline conditions between the 2 groups (*P* > .05), indicating a harmonious and comparable baseline. Refer to Table 2 for a detailed overview.

**Table 2:** Characteristics of participants in test group and control group.

	Test group	Control group	*P* value
Number	35	37	
Age (years)	42.1 ± 11.0	43.81 ± 9.3	.49
Height (cm)	162 (158–165)	162 (160–167)	.55
Weight (kg)	58.0 (52.0–67.0)	60 (50.50–68.50)	.74
Dose of levothyroxine sodium tablets (µg/d)	87.5 (75–112.5)	75.0 (62.5–100.0)	.28
Medical insurance			.23
Self-financed patient	7 (20.00%)	12 (32.43%)	
Medicare patient	28 (80.00%)	25 (67.57%)	
Gender			.60
Male	5 (14.29%)	7 (18.92%)	
Female	30 (85.71%)	30 (81.08%)	
Occupation			.97
Self-employed	5 (14.29%)	6 (16.22%)	
Employee	16 (45.71%)	18 (48.65%)	
Retired	6 (17.14%)	6 (16.22%)	
Others	8 (22.86%)	7 (18.92%)	
Education			.48
High school and below	16 (45.71%)	20 (54.05%)	
Junior college and above	19 (54.29%)	17 (45.95%)	
Marital status			.93
Unmarried	4 (11.43%)	4 (10.81%)	
Married	31 (88.57%)	33 (89.19%)	
Fertility status			.28
Having babies	7 (20.00%)	4 (10.81%)	
Not having babies	28 (80.00%)	33 (89.19%)	
Gene mutation			.66
Not detected	26 (74.29%)	24 (64.86%)	
BRAFV600e mutation	8 (22.86%)	12 (32.435)	
Other types of mutations	1 (2.86%)	1 (2.70%)	
Lymphatic metastasis			.97
No	20 (57.14%)	21 (56.76%)	
Yes	15 (42.86%)	16 (43.24%)	
Cancer stage*			.33
I	34 (97.14%)	34 (91.89%)	
II	1 (2.86%)	3 (8.11%)	
I^131^ treatment			.82
No	30 (85.71%)	31 (83.78%)	
Yes	5 (14.29%)	6 (16.22%)	
HAMD score stratification			.93
8 ≤ Score < 20	30 (85.71%)	32 (86.49%)	
Score ≥ 20	5 (14.29%)	5 (13.51%)	

HAMD = Hamilton Depression Scale.

* Clinical stage classified on the basis of on the American Joint Committee on Cancer 7^th^ edition staging system.

### 3.2. Depression assessment and efficacy

**Figure 2.**
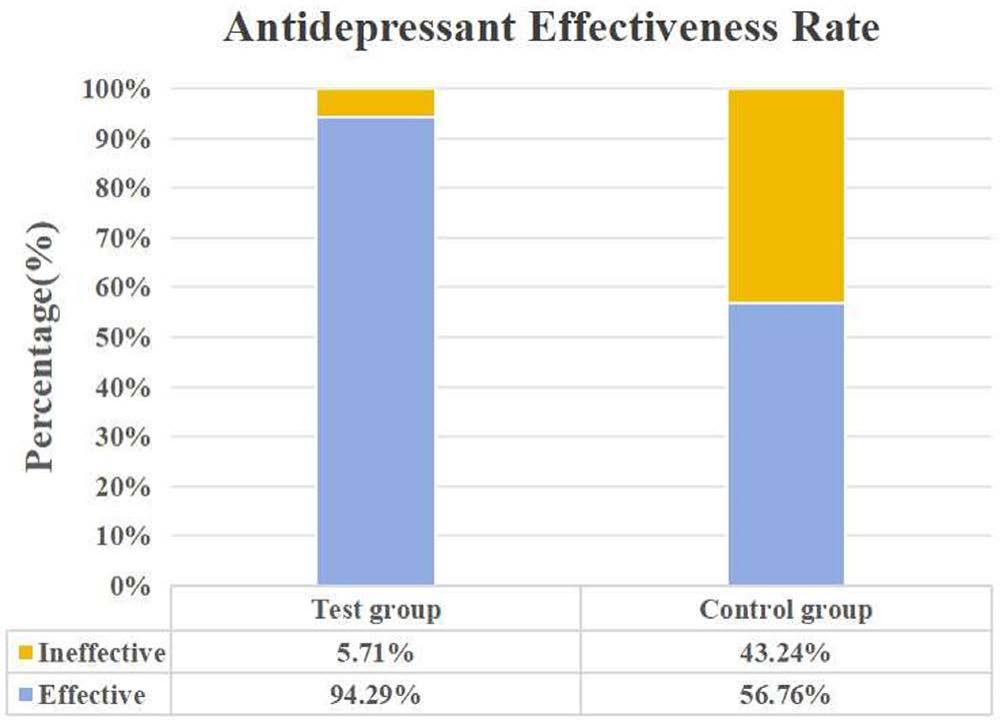
The antidepressant effectiveness rate in the test and control groups.

The antidepressant effectiveness rate of the test and control groups demonstrated 94.29% and 56.76%, respectively (Fig. 2). Prior to treatment initiation, no statistically significant differences were observed in HAMD scores and HAMD-related factor scores between the 2 groups. Following the intervention, the test group exhibited a significant reduction in both HAMD scores and HAMD-related factor scores, including anxiety/somatization, cognitive disorder, sleep disorder, hopelessness, and retardation (*P* < .05). Similarly, the control group experienced a significant decrease in HAMD scores and HAMD-related factor scores (anxiety/somatization, cognitive disorder, sleep disorder, hopelessness, and retardation) compared to the pretreatment period (*P* < .05). Comparative analysis between the test and control groups revealed significant reductions in HAMD score and HAMD-related factor scores (anxiety/somatization, sleep disorder, and hopelessness) in the test group compared to the control group (*P* < .05). Refer to Table 3 for a comprehensive presentation of these findings.

**Table 3:** Analysis of HAMD scores and associated factor scores.

Item	Groups	Duration of treatment (wk)	Scores	*P* value between groups		*P* value within group	
				Weeks 0	Weeks 12	Test group	Control group
Total scores	Test group (N = 35)	Weeks 0	14.00 ± 4.25	.93	<.01	<.01	<.01
		Weeks 12	7.23 ± 2.35				
	Control group (N = 37)	Weeks 0	14.03 ± 4.05				
		Weeks 12	10.05 ± 3.44				
Anxiety/Somatization	Test group (N = 35)	Weeks 0	6.23 ± 1.70	.49	<.05	<.01	<.01
		Weeks 12	4.17 ± 0.98				
	Control group (N = 37)	Weeks 0	6.46 ± 1.59				
		Weeks 12	4.97 ± 1.36				
Weight	Test group (N = 35)	Weeks 0	0.14 ± 0.36	.96	.79	.94	.33
		Weeks 12	0.14 ± 0.49				
	Control group (N = 37)	Weeks 0	0.22 ± 0.58				
		Weeks 12	0.14 ± 0.42				
Cognitive disorder	Test group (N = 35)	Weeks 0	1.71 ± 1.02	.08	.10	<.01	<.01
		Weeks 12	0.40 ± 0.55				
	Control group (N = 37)	Weeks 0	1.30 ± 1.18				
		Weeks 12	0.76 ± 0.86				
Diurnal variation	Test group (N = 35)	Weeks 0	0.26 ± 0.24	.59	.22	.13	.79
		Weeks 12	0.11 ± 0.40				
	Control group (N = 37)	Weeks 0	0.24 ± 0.55				
		Weeks 12	0.22 ± 0.48				
Retardation	Test group (N = 35)	Weeks 0	1.43 ± 0.92	.80	.15	<.01	<.05
		Weeks 12	0.86 ± 0.65				
	Control group (N = 37)	Weeks 0	1.49 ± 0.99				
		Weeks 12	1.11 ± 0.74				
Sleep disorder	Test group (N = 35)	Weeks 0	2.17 ± 1.58	.45	<.05	<.01	<.05
		Weeks 12	1.11 ± 1.35				
	Control group (N = 37)	Weeks 0	2.49 ± 1.48				
		Weeks 12	1.89 ± 1.39				
Hopelessness	Test group (N = 35)	Weeks 0	2.06 ± 1.03	.48	<.01	<.01	<.01
		Weeks 12	0.43 ± 0.65				
	Control group (N = 37)	Weeks 0	1.84 ± 1.01				
		Weeks 12	0.95 ± 0.88				

HAMD = Hamilton Depression Scale.

### 3.3. Serum 5-HT concentrations in 2 groups

pretreatment, there existed no significant disparity in serum 5-HT levels between the 2 groups. Post-treatment, the test group exhibited a substantial elevation in serum 5-HT levels compared to the pretreatment period (*P* < .05). Conversely, the control group demonstrated no significant variance in serum 5-HT levels when compared to the pretreatment period. Moreover, the serum 5-HT levels in the test group were significantly higher than those in the control group (*P* < .05), as detailed in Table 4.

**Table 4:** Analysis of 5-HT concentrations in serum.

	Weeks 0 5-HT (nm/L)	Weeks 12 5-HT (nm/L)	*P* value within group
Test group (N = 35)	278.48 ± 140.35	321.02 ± 84.40	<.05
Control group (N = 37)	296.75 ± 115.82	276.06 ± 91.90	.20
*P* value between groups	.24	<.05	

### 3.4. HRQoL analysis in 2 groups

Prior to treatment initiation, no significant differences were discerned in the scores of the 2 groups across domains encompassing functional scales, symptom sub-scales, and global health status. Post-treatment, the test group exhibited noteworthy improvements, with scores reflecting elevated emotional functioning, social functioning, and global health status, alongside significantly lower scores in fatigue and insomnia compared to the pretreatment period (*P* < .05). Similarly, the control group experienced significant enhancements, with increased scores in social functioning and global health status, and decreased scores in fatigue and insomnia compared to the pretreatment period (*P* < .05). A statistical difference was only observed between the test and control groups in the emotional domain (*P* < .05), as illustrated in Table 5.

**Table 5:** Analysis of HRQoL in 2 groups.

	Groups	Duration of treatment (wk)	Scores	*P* value between groups		*P* value within group	
				Weeks 0	Weeks 12	Test group	Control group
Physical functioning	Test group (N = 35)	Weeks 0	88.19 ± 11.21	.25	.07	.76	.49
		Weeks 12	88.76 ± 9.26				
	Control group (N = 37)	Weeks 0	85.77 ± 11.13				
		Weeks 12	85.04 ± 9.61				
Role functioning	Test group (N = 35)	Weeks 0	88.57 ± 15.00	.91	.78	.09	.05
		Weeks 12	92.86 ± 12.32				
	Control group (N = 37)	Weeks 0	88.29 ± 15.14				
		Weeks 12	92.34 ± 12.17				
Emotional functioning	Test group (N = 35)	Weeks 0	65.24 ± 22.46	.34	.02	<.01	.65
		Weeks 12	79.05 ± 15.57				
	Control group (N = 37)	Weeks 0	70.05 ± 18.89				
		Weeks 12	70.27 ± 14.51				
Cognitive functioning	Test group (N = 35)	Weeks 0	77.14 ± 19.84	.53	.81	.51	.49
		Weeks 12	80.00 ± 20.93				
	Control group (N = 37)	Weeks 0	77.48 ± 14.81				
		Weeks 12	80.63 ± 17.35				
Social functioning	Test group (N = 35)	Weeks 0	84.29 ± 18.50	.76	.17	<.01	.01
		Weeks 12	93.81 ± 12.18				
	Control group (N = 37)	Weeks 0	81.08 ± 23.62				
		Weeks 12	89.64 ± 14.35				
Global health status	Test group (N = 35)	Weeks 0	70.48 ± 10.57	.63	.10	<.01	.01
		Weeks 12	79.76 ± 8.15				
	Control group (N = 37)	Weeks 0	68.92 ± 14.39				
		Weeks 12	74.32 ± 13.94				
Fatigue	Test group (N = 35)	Weeks 0	31.75 ± 20.19	.83	.50	<.01	<.01
		Weeks 12	18.73 ± 16.12				
	Control group (N = 37)	Weeks 0	33.93 ± 21.90				
		Weeks 12	22.22 ± 20.45				
Nausea and vomiting	Test group (N = 35)	Weeks 0	11.43 ± 13.27	.36	.10	.07	.06
		Weeks 12	5.71 ± 10.65				
	Control group (N = 37)	Weeks 0	13.97 ± 12.74				
		Weeks 12	9.46 ± 11.48				
Pain	Test group (N = 35)	Weeks 0	1.90 ± 7.85	.30	.29	1.00	.91
		Weeks 12	1.90 ± 7.85				
	Control group (N = 37)	Weeks 0	3.15 ± 8.64				
		Weeks 12	4.05 ± 12.67				
Dyspnea	Test group (N = 35)	Weeks 0	12.38 ± 21.52	.45	.27	.80	.20
		Weeks 12	11.42 ± 16.05				
	Control group (N = 37)	Weeks 0	14.41 ± 18.49				
		Weeks 12	8.11 ± 16.49				
Insomnia	Test group (N = 35)	Weeks 0	32.38 ± 29.69	.61	.11	<.01	<.01
		Weeks 12	14.28 ± 20.27				
	Control group (N = 37)	Weeks 0	36.04 ± 28.74				
		Weeks 12	22.52 ± 23.64				
Appetite loss	Test group (N = 35)	Weeks 0	8.57 ± 14.78	.54	.44	.74	.21
		Weeks 12	7.62 ± 16.34				
	Control group (N = 37)	Weeks 0	7.21 ± 15.98				
		Weeks 12	4.50 ± 11.55				
Constipation	Test group (N = 35)	Weeks 0	9.52 ± 15.28	.21	.41	.10	.08
		Weeks 12	4.76 ± 11.83				
	Control group (N = 37)	Weeks 0	5.40 ± 12.45				
		Weeks 12	2.70 ± 9.22				
Diarrhea	Test group (N = 35)	Weeks 0	7.62 ± 18.23	.38	.40	.39	.48
		Weeks 12	4.76 ± 14.33				
	Control group (N = 37)	Weeks 0	9.91 ± 17.33				
		Weeks 12	7.21 ± 15.98				
Financial difficulties	Test group (N = 35)	Weeks 0	6.67 ± 13.53	.68	.38	.08	.32
		Weeks 12	3.81 ± 10.76				
	Control group (N = 37)	Weeks 0	5.40 ± 12.45				
		Weeks 12	6.31 ± 13.23				

### 3.5. Qi-Yin deficiency assessment and efficacy

**Figure 3.**
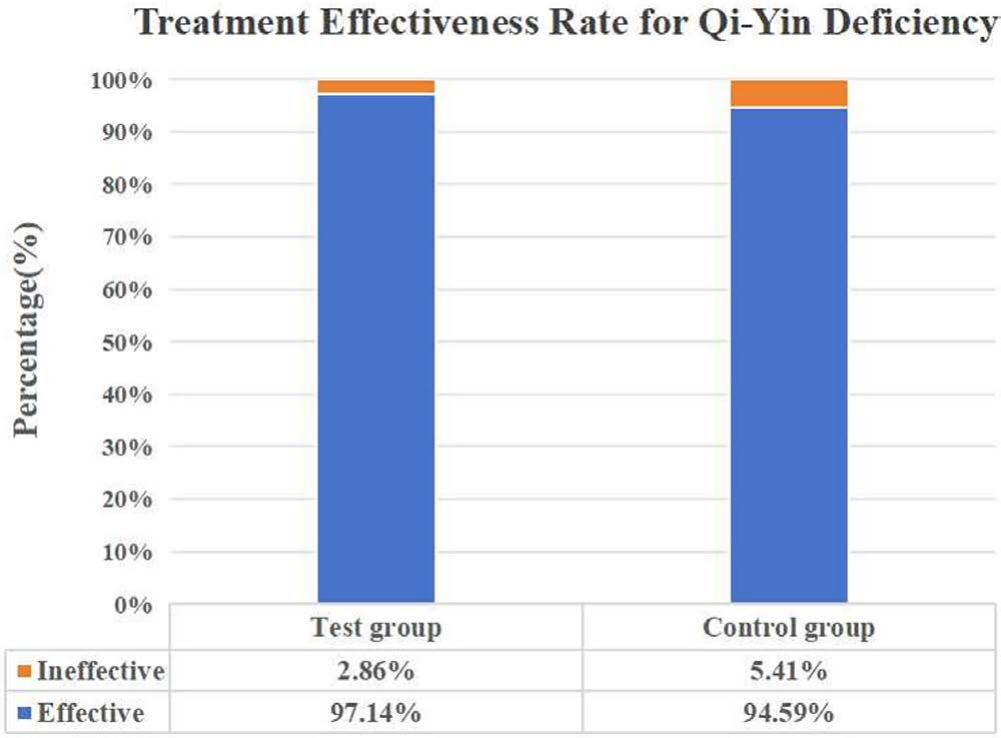
The TCM treatment effectiveness rate for Qi-Yin deficiency in the test and control groups. TCM = traditional Chinese medicine.

The TCM treatment for Qi-Yin deficiency showcased efficacy in both the test and control groups, with an effective rate of 97.14% and 94.59%, respectively (Fig. 3). Prior to treatment initiation, there existed no statistically significant difference in Qi-Yin deficiency syndrome scores between the 2 groups. Post-treatment, both the test and control groups demonstrated a significant decrease in Qi-Yin deficiency syndrome scores compared to the pretreatment period (*P* < .01). Importantly, there was no significant difference observed in the Qi-Yin deficiency syndrome scores between the test and control groups, as detailed in Table 6.

**Table 6:** Analysis of Qi-Yin deficiency syndrome scores.

	Weeks 0	Weeks 12	*P* value within group
Test group (N = 35)	19.54 ± 5.47	5.94 ± 3.09	<.01
Control group (N = 37)	19.73 ± 4.97	5.59 ± 3.43	<.01
*P* value between groups	.88	.69	

### 3.6. Inflammatory factors analysis in 2 groups

pretreatment, no significant differences were observed in inflammatory factors between the test and control groups. Post-treatment, there remained no significant difference in inflammatory factors between the 2 groups. However, McNemar’s test results revealed a substantial increase in the IFN-γ < 2.44 rate (62.86%) in the test group compared to those (25.71%) of pretreatment period after treatment (*P* < .05). Similarly, the IL-6 < 2.44 rate (74.29%) exhibited a significant increase compared to those (48.57%) of pretreatment period after treatment (*P* < .05). Conversely, the control group demonstrated no significant difference in inflammatory factors before and after treatment. For a detailed overview, refer to Table 7.

**Table 7:** Analysis of inflammatory factor.

	Range	Weeks 0		Weeks 12		*P* value between groups		*P* value within groups	
		Test group (N = 35)	Control group (N = 37)	Test group (N = 35)	Control group (N = 37)	Weeks 0	Weeks 12	Test group	Control group
IFN-γ (pg/mL)	<2.44	9 (25.71%)	13 (35.14%)	22 (62.86%)	19 (51.35%)	.39	.32	<.05	.24
	≥2.44	26 (74.29%)	24 (64.86%)	13 (37.14%)	18 (48.65%)				
IL-1β (pg/mL)	<2.44	25 (71.43%)	31 (83.78%)	32 (91.43%)	31 (83.78%)	.21	.33	.07	1.00
	≥2.44	10 (28.57%)	6 (16.22%)	3 (8.57%)	6 (16.22%)				
IL-6 (pg/mL)	<2.44	17 (48.57%)	23 (62.16%)	26 (74.29%)	26 (70.27%)	.25	.70	<.05	.58
	≥2.44	18 (51.43%)	14 (37.84%)	9 (25.71%)	11 (29.73%)				
IL-8 (pg/mL)	<2.44	21 (60.00%)	26 (70.27%)	29 (82.86%)	29 (78.38%)	.36	.63	.06	.51
	≥2.44	14 (40.00%)	11 (29.73%)	6 (17.14%)	8 (21.62%)				
TNF-α (pg/mL)	<2.44	25 (71.43%)	27 (72.97%)	28 (80.00%)	32 (86.49%)	.88	.46	.51	.23
	≥2.44	10 (28.57%)	10 (27.03%)	7 (20.00%)	5 (13.51%)				

IFN-γ = interferon gamma, IL-1β = interleukin-1β, IL-6 = interleukin-6, IL-8 = interleukin-8, TNF-α = tumor necrosis factor α.

### 3.7. Thyroid function analysis

Both before and after treatment, no noteworthy differences were discerned in thyroid function indices between the test and control groups. Furthermore, within-group comparisons for both the test and control groups did not reveal significant variations in the thyroid function indices, as detailed in Table 8.

**Table 8:** Thyroid function.

	Weeks 0		Weeks 12		*P* value between groups		*P* value within groups	
	Test group (N = 35)	Control group (N = 37)	Test group (N = 35)	Control group (N = 37)	Weeks 0	Weeks 12	Test group	Control group
TSH mIU/L	1.50 ± 2.85	1.36 ± 1.41	1.09 ± 1.84	1.25 ± 1.90	.60	.76	.26	.27
T3 ng/mL	1.10 ± 0.33	1.13 ± 0.25	1.04 ± 0.22	1.10 ± 0.17	.26	.05	.39	.52
T4 nmol/L	111.81 ± 22.30	119.38 ± 23.78	111.80 ± 20.74	114.76 ± 19.59	.09	.54	.85	.13
Tg ng/mL								
<10	30 (85.71%)	34 (91.89%)	32 (91.43%)	35 (94.59%)	.40	.60	.50	1.00
≥10	5 (14.29%)	3 (8.11%)	3 (8.57%)	2 (5.41%)				
TgAb IU/mL								
<10	9 (25.71%)	10 (27.03%)	9 (25.71%)	7 (18.92%)	.90	.49	1.00	.55
≥10	26 (74.29%)	27 (72.97%)	26 (74.29%)	30 (81.08%)				

T3 = triiodothyronine, T4 = thyroxine, Tg = thyroglobulin, TgAb = anti-thyroglobulin antibodies, TSH = thyroid stimulating hormone.

## 4. Discussion

Aligned with established guidelines, this study endeavored to assess the clinical efficacy of the soothing Liver-Qi stagnation method on P-PTC patients presenting Qi-Yin deficiency syndrome accompanied by depression, employing a clinical randomized controlled trial. The results underscored the antidepressant efficacy of the test group at 94.29%, the control group’s rate of 56.76%. Subsequent to treatment, HAMD scores exhibited a significant decrease in the test group compared to the control group (*P* < .05), particularly in anxiety/somatization, sleep disorder, and hopelessness factors (*P* < .05). Additionally, post-treatment serum 5-HT levels were significantly higher in the test group than the control group (*P* < .05). The TCM treatment for Qi-Yin deficiency showcased efficacy in both groups, with the test group at 97.14% and the control group at 94.59%. Notably, TCM treatment efficacy significantly decreased post-treatment compared to pretreatment (*P* < .05), with no significant difference observed in Qi-Yin deficiency scores between the 2 groups after treatment. Scores from the EORTC QLQ-C30 V3.0 Chinese version exhibited improved social function and global health status, and reduced fatigue and insomnia symptoms post-treatment compared to baseline (*P* < .05). However, emotional domain scores were significantly higher in the test group compared to the control group (*P* < .05). In the analysis of inflammatory factors, no significant post-treatment differences were observed between the 2 groups. Noteworthy increases were found in IFN-γ < 2.44 rate (62.86%) and IL-6 < 2.44 rate (74.29%) in the test group compared to pretreatment levels (*P* < .05). Conversely, the control group showed no significant disparity between pretreatment and post-treatment inflammatory factors. In conclusion, the Soothing Liver-Qi stagnation method exhibited promising results in ameliorating depressive symptoms and improving the quality of life among P-PTC presenting Qi-Yin deficiency syndrome.

The incidence of thyroid cancer is increasing, and although post-PTC patients have a good prognosis, with a survival rate of 95% at 20 years, however, their quality of life and psychosocial situation is much lower than that of the normal population.^[19]^ Postoperative patients with thyroid cancer often have a large psychological burden due to the fear of recurrence, which is mostly manifested as depression.^[7]^ Both medication and psychotherapy are effective in treating depressive symptoms in cancer patients.^[35]^Our previous study showed that CHM demonstrated some benefit in the treatment of concomitant depression in oncology patients.^[15]^ CHM therapy is not only a kind of medication, but also a kind of psychotherapy, belonging to the cognitive behavioral therapy in psychotherapy.^[15]^ Within the framework of TCM theory, “Liver qi stagnation” is identified as a primary contributor to depression, and the method of soothing Liver-Qi stagnation has proven effective in its treatment.^[22]^ Clinically, the compound CH-BS, commonly utilized to treat depression through the soothing Liver-Qi stagnation method.^[25]^ Studies have highlighted the efficacy of TCM formulas containing antidepressant herbs in reducing mortality associated with prostate cancer comorbid depression.^[36]^ When tumor patients are accompanied by depression, antitumor and antidepressant treatments are administered concurrently. Thus in our study, the inclusion of CH-BS in the decoction of benefiting qi and nourishing yin not only guarantees anti-tumor efficacy but also acts as an antidepressant, thereby serving dual therapeutic objectives concurrently.

In light of the escalating global incidence of thyroid cancer, an increasing number of scholars have turned their attention to the emotional well-being of P-PTC patients. Lv et al^[37]^ conducted a comprehensive assessment of the depressive state in P-PTC patients using the depression self-assessment scale. Their findings revealed a pervasive presence of depressive symptoms in this patient population. Nuria et al,^[38]^ employing the hospital anxiety and depression scale, similarly identified a high prevalence of depression among P-PTC patients. In this current investigation, we utilized the HAMD to evaluate the presence of depression in P-PTC patients. Our results align with prior studies, indicating a prevalent manifestation of depression in this cohort. Notably, existing scales for assessing depression in cancer patients lack universal standardization. The Hamilton Depression Scale, a widely accepted tool for evaluating depression, exhibits high specificity for depressive symptoms.^[39]^ The selection of HAMD as the primary instrument for evaluating cancer-related depression was on account of its prominence in the field.^[40]^ Consequently, in this study, we selected the HAMD scale to conduct an in-depth analysis of depression-related factor scores in P-PTC patients. Notably, these factors primarily manifested as anxiety/somatization, cognitive impairment, sleep disturbance, retardation, and hopelessness. Existing evidence suggests that patients with thyroid cancer face a heightened risk of cognitive impairment, potentially influenced by alterations in cognitive processes stemming from TSH levels, cytokines, and immune cell activity.^[41]^ Concurrently, P-PTC patients often grapple with a confluence of anxiety and depressive states.^[7,42]^ Sleep disorders observed in this cohort align with circadian rhythm disruption – a cardinal feature of depression. Notably, phenomena such as early awakening and heightened susceptibility to arousal characterize this sleep disturbance.^[43]^ In the current investigation, our assessment utilizing the HAMD Scale uncovered that the application of the soothing Liver-Qi stagnation method effectively ameliorated associated depressive symptoms, including anxiety, cognitive disorder, retardation, sleep disturbance, and hopelessness, in P-PTC patients. Remarkably, outcomes within the control group indicated that herbs promoting qi and nourishing yin also contributed to improvements in depressive states, as evidenced by reduced HAMD scale scores and associated factors (anxiety, cognitive disorder, retardation, sleep disorder, and hopelessness) when compared to the pretreatment period. This aligns harmoniously with our prior research, affirming the depression alleviation potential of CHM treatments in the context of tumor patients.^[15]^ However, these scores remained significantly higher than those observed in the test group. Notably, the application of the soothing Liver-Qi stagnation method emerged as particularly efficacious, though further support from rigorous clinical investigations is warranted to substantiate these observations.

Serum concentrations of 5-HT have demonstrated a robust association with depression.^[18]^ In light of related studies, it has been elucidated that the inhibition of 5-HT reuptake and elevation of extracellular 5-HT concentration can effectively serve an antidepressant role.^[44]^ TCM has been documented to exert its anti-depression effects through the modulation of monoamine neurotransmitter levels, the regulation of inflammatory responses, influence on the hypothalamic-pituitary-adrenal axis hormones, and modulation of neurotrophic factors.^[24]^ An et al in a study treating patients with cancer-related depression, employed a combination of Chaihu plus Longgu Muli decoction in conjunction with 5-element music therapy. The results revealed a significant elevation in serum 5-HT levels among treated individuals, accompanied by a tangible alleviation of cancer-related depression symptoms.^[40]^ In our investigation, we observed a noteworthy outcome where the adjunctive use of CH-BS in the decoction focused on benefiting qi and nourishing yin played a pivotal role in mitigating Liver-Qi stagnation. This intervention led to a discernible increase in serum concentrations of 5-HT among P-PTC patients, consequently contributing to the alleviation of depression.

Despite the more favorable prognosis observed in P-PTC patients, their diminished quality of life does not diverge from the compromised quality of life experienced by patients with other malignancies.^[41]^ In investigation, we assessed the quality of life of patients in both the test and control groups using the EORTC QLQ-C30 V3.0 Chinese version of the Quality of Life Scale. Encouragingly, our results demonstrated a notable enhancement in the quality of life for both groups following treatment. Consistent with existing literature, Traditional Chinese Medicine (TCM) interventions have exhibited the ability to augment both the quality of life and survival rates among cancer patients, findings substantiated by several studies.^[45,46]^ Fatigue is a frequently observed symptom in P-PTC patients, potentially attributable to thyroid hormone suppression.^[47]^ In our study, notable reductions in fatigue symptom scores were observed in both groups post-treatment, aligning with findings from related studies validating the efficacy of alleviating fatigue symptoms in oncology patients.^[15]^ Interestingly, the test group, augmented with CH-BS, exhibited a distinctive profile relative to the control group, particularly evident in emotional functioning. This disparity may underscore the nuanced impact of CH-BS in influencing emotional well-being, contributing to a significant difference observed between the 2 groups in this specific domain.

The concept of syndrome stands as a pivotal bridge in TCM, serving as the linchpin for both syndrome differentiation and subsequent therapeutic interventions. In adherence to TCM principles, practitioners meticulously align their prescriptions with the specific TCM syndrome identified. Noteworthy Chinese studies have elucidated that cancer patients experiencing mild-to-moderate depression predominantly exhibit Qi-Yin deficiency syndromes.^[48]^ Aligned with this insight, our study focused on individuals P-PTC patients who exhibited Qi-Yin deficiency syndrome as the primary observational cohort. Our results underscored a consistent manifestation of depression across all included patients with Qi-Yin deficiency syndromes. In order to gauge the efficacy of basic herbs in addressing Qi-Yin deficiency syndrome, we employed the Qi-Yin deficiency syndrome questionnaire. Encouragingly, both groups exhibited significant reductions in syndrome scores post-treatment compared to their pretreatment baseline.

In recent years, advancements in understanding the neurobiology of depression and the physiopathology of cancer have brought to light shared biological mechanisms. Research indicates that both depression and cancer are intricately associated with inflammation and immune system dysregulation.^[49]^ Extensive research has demonstrated a close relationship between inflammatory factors, such as IFN-γ, IL-6, IL-8, IL-1β, and TNF-α, and the development of both depression and tumors.^[39,50]^ These inflammatory factors, particularly IFN-γ and IL-6, exert an immunosuppressive effect on immune cells, leading to a reduction in T-lymphocyte activity, thereby contributing to the persistence of depression.^[51]^ Multiple studies have also reported elevated levels of inflammatory factors, including IFN-γ, IL-6, IL-8, IL-1β, and TNF-α, in the blood of individuals experiencing depression.^[49,52]^ In the context of our study, we conducted assessments of inflammatory factors in patients before and after treatment. The results revealed significant changes in IFN-γ and IL-6 levels within the test group, indicating that the soothing Liver-Qi stagnation method effectively mitigates inflammatory factors associated with depression.

All P-PTC patients received treatment with levothyroxine sodium tablets, ensuring relative stability in thyroid-related hormone indexes before and after treatment. This approach helps mitigate potential analysis bias arising from the instability of relevant variables.

## 5. Conclusion

In conclusion, our study demonstrated that the soothing Liver-Qi stagnation method, employed in a randomized controlled clinical trial, effectively mitigated depression in P-PTC patients presenting with Qi-Yin deficiency syndrome. This method exhibited antidepressant effects by elevating serum levels of 5-HT and reducing inflammatory factors associated with depression. While our findings are promising, it is crucial to acknowledge certain limitations within this study, notably the relatively small sample size. In subsequent investigations, we aim to expand the sample size to comprehensively validate the long-term clinical efficacy of the soothing Liver-Qi stagnation method. This effort seeks to provide a robust, scientific, and reliable evidence-based foundation for the clinical application of this method in managing depression among P-PTC patients, thus contributing to the advancement of therapeutic strategies in this patient population.

## Acknowledgments

We would like to thank all the patients who participated in this study for their support and understanding of our work. We acknowledge the Scientific Research Office of Longhua Hospital Shanghai University of Traditional Chinese Medicine, Shanghai, China for supporting our study (no. KY1938).

## Author contributions

**Conceptualization:** Huiyue Lin, Juyong Wang

**Data curation:** Huiyue Lin, Xueting Zhang

**Formal analysis:** Huiyue Lin, Xueting Zhang, Chenchen Tang

**Investigation:** Huiyue Lin, Xueting Zhang, Yuqian Zheng, Chenchen Tang

**Methodology:** Yuqian Zheng, Chenchen Tang

**Software:** Yuqian Zheng

**Supervision:** Juyong Wang

**Writing – original draft:** Huiyue Lin

**Writing – review & editing:** Juyong Wang

## References

[1] Maomao C He L Dianqin S. Current cancer burden in China: epidemiology, etiology, and prevention. Cancer Biol Med. 2022;19:1121–38. 36069534 10.20892/j.issn.2095-3941.2022.0231 PMC9425189

[2] Chen W Zheng R Baade PD. Cancer statistics in China, 2015. CA Cancer J Clin. 2016;66:115–32. 26808342 10.3322/caac.21338

[3] Helgeson VS Cohen S Schulz R Yasko J. Group support interventions for women with breast cancer: who benefits from what. Health Psychol. 2000;19:107–14. 10762094 10.1037//0278-6133.19.2.107

[4] Goswami S Mongelli M Peipert BJ Helenowski I Yount SE Sturgeon C. Benchmarking health-related quality of life in thyroid cancer versus other cancers and United States normative data. Surgery. 2018;164:986–92. 30149935 10.1016/j.surg.2018.06.042

[5] Hedman C Strang P Djärv T Widberg I Lundgren CI. Anxiety and fear of recurrence despite a good prognosis: an interview study with differentiated thyroid cancer patients. Thyroid. 2017;27:1417–23. 28874092 10.1089/thy.2017.0346

[6] Dong W Horiuchi K Tokumitsu H. Time-varying pattern of mortality and recurrence from papillary thyroid cancer: lessons from a long-term follow-up. Thyroid. 2019;29:802–8. 30931815 10.1089/thy.2018.0128

[7] Yang S Wang J Xu X. Psychological health status among thyroid cancer patients during the COVID-19 epidemic in China. Support Care Cancer. 2022;30:2111–9. 34671859 10.1007/s00520-021-06624-9 PMC8528476

[8] Zhu GL Xu C Yang KB. Causal relationship between genetically predicted depression and cancer risk: a two-sample bi-directional mendelian randomization. BMC Cancer. 2022;22:353. 35361153 10.1186/s12885-022-09457-9 PMC8973550

[9] Su YR Yu XP Huang LQ Xie L Zha JS. Factors influencing postoperative anxiety and depression following Iodine-131 treatment in patients with differentiated thyroid cancer: a cross-sectional study. World J Psychiatry. 2023;13:486–94. 37547735 10.5498/wjp.v13.i7.486 PMC10401505

[10] Vujovic D Alsen M Vasan V Genden E van Gerwen M. anxiety and depression as potential predictors for shorter time to undergo initial surgical treatment for papillary thyroid cancer. Cancers (Basel). 2024;16:545. 38339296 10.3390/cancers16030545 PMC10854873

[11] Mitchell AL Gandhi A Scott-Coombes D Perros P. Management of thyroid cancer: United Kingdom National Multidisciplinary Guidelines. J Laryngol Otol. 2016;130:S150–60. 27841128 10.1017/S0022215116000578 PMC4873931

[12] Haugen BR Alexander EK Bible KC. American Thyroid Association Management Guidelines for adult patients with thyroid nodules and differentiated thyroid cancer. Thyroid. 20152015;26:1–133. 10.1089/thy.2015.0020 PMC4739132 26462967

[13] Tufekci D Ayaz T Sahin SB Hocaoglu C. The “Non-Treated” Versus “LT3-Treated” protocols of short-term hypothyroidism induction in differentiated thyroid cancer: an analysis of hypothyroid complications, mood disorders, and quality of life. Horm Metab Res. 2023;55:462–70. 37059443 10.1055/a-2056-6073

[14] Dayan CM Panicker V. Hypothyroidism and depression. Eur Thyroid J. 2013;2:168–79. 24847450 10.1159/000353777 PMC4017747

[15] Lin H Zhang X Zhang Y Cui W Jia F Wang J. Association between Chinese herbal medicine (CHM) treatment and depression among cancer patients in China: an outpatient-based cross-sectional study. Medicine (Baltimore). 2023;102:e34695. 37653736 10.1097/MD.0000000000034695 PMC10470751

[16] Sarrouilhe D Clarhaut J Defamie N Mesnil M. Serotonin and cancer: what is the link? Curr Mol Med. 2015;15:62–77. 25601469 10.2174/1566524015666150114113411

[17] Tamir H Hsiung SC Yu PY. Serotonergic signalling between thyroid cells: protein kinase C and 5-HT2 receptors in the secretion and action of serotonin. Synapse. 1992;12:155–68. 1336223 10.1002/syn.890120209

[18] Kraus C Castrén E Kasper S Lanzenberger R. Serotonin and neuroplasticity – links between molecular, functional and structural pathophysiology in depression. Neurosci Biobehav Rev. 2017;77:317–26. 28342763 10.1016/j.neubiorev.2017.03.007

[19] Mols F Schoormans D Netea-Maier R. Determinants and mediating mechanisms of quality of life and disease-specific symptoms among thyroid cancer patients: the design of the WaTCh study. Thyroid Res. 2023;16:23. 37424010 10.1186/s13044-023-00165-5 PMC10332086

[20] Comai S Nunez N Atkin T. Dysfunction in endocannabinoids, palmitoylethanolamide, and degradation of tryptophan into kynurenine in individuals with depressive symptoms. BMC Med. 2024;22:33. 38273283 10.1186/s12916-024-03248-8 PMC10809514

[21] Wang ZQ Zhou Y Guan QQ Xia ZY. TCM patterns types and indicators of thyroid cancer by Delphi method: an investigation. J Beijing Univ Tradit Chin Med. 2016;39:955–960964.

[22] Chen Y Wang W Fu X. Investigation of the antidepressant mechanism of combined Radix Bupleuri and Radix Paeoniae Alba treatment using proteomics analysis of liver tissue. J Chromatogr B Analyt Technol Biomed Life Sci. 2021;1179:122858. 10.1016/j.jchromb.2021.122858 34329891

[23] Wang S Long S Deng Z Wu W. Positive role of Chinese herbal medicine in cancer immune regulation. Am J Chin Med. 2020;48:1577–92. 33202152 10.1142/S0192415X20500780

[24] Wang YS Shen CY Jiang JG. Antidepressant active ingredients from herbs and nutraceuticals used in TCM: pharmacological mechanisms and prospects for drug discovery. Pharmacol Res. 2019;150:104520. 31706012 10.1016/j.phrs.2019.104520

[25] Zhang F Zhou K Yuan W Sun K. Radix Bupleuri-Radix Paeoniae Alba inhibits the development of hepatocellular carcinoma through activation of the PTEN/PD-L1 Axis within the Immune Microenvironment. Nutr Cancer. 2024;76:63–79. 37909316 10.1080/01635581.2023.2276525

[26] National Health Commission of the People’s Republic of China. Thyroid Cancer Diagnostic and Treatment Guidelines (2018 Edition). Chin Arch Gen Surg (Electronic Edition). 2019;13:1–15.

[27] World Health Organization. ICD-11 revision. https://icd.who.int/en. Accessed June 18, 2020.

[28] Cui Y Zhang H Wang S. Stimulated parotid saliva is a better method for depression prediction. Biomedicines. 2022;10:2220. 36140321 10.3390/biomedicines10092220 PMC9496557

[29] Zheng X. Guiding Pricinples for Traditional Chinese Medicine and New Medicine on Clinical Trials. 1st ed. China Medical Science Press (Beijing); 2002.

[30] Hamilton MA. rating scale for depression. J Neurol Neurosurg Psychiatry. 1960;23:56–62. 14399272 10.1136/jnnp.23.1.56 PMC495331

[31] Dai L Wang P Du H. High-frequency Repetitive Transcranial Magnetic Stimulation (rTMS) accelerates onset time of beneficial treating effects and improves clinical symptoms of depression. CNS Neurol Disord Drug Targets. 2022;21:500–10. 34736388 10.2174/1871527320666211104123343

[32] Aaronson NK Ahmedzai S Bergman B. The European Organization for Research and Treatment of Cancer QLQ-C30: a quality-of-life instrument for use in international clinical trials in oncology. J Natl Cancer Inst. 1993;85:365–76. 8433390 10.1093/jnci/85.5.365

[33] Zhao H Kanda K. Testing psychometric properties of the standard Chinese version of the European Organization for Research and Treatment of Cancer Quality of Life Core Questionnaire 30 (EORTC QLQ-C30). J Epidemiol. 2004;14:193–203. 15617393 10.2188/jea.14.193 PMC8784239

[34] Eberst G Anota A Scherpereel A.; French Cooperative Thoracic Intergroup (IFCT). Health-related quality of life impact from adding bevacizumab to cisplatin-pemetrexed in malignant pleural mesothelioma in the MAPS IFCT-GFPC-0701 Phase III Trial. Clin Cancer Res. 2019;25:5759–65. 31175096 10.1158/1078-0432.CCR-18-2860

[35] Akechi T Furukawa TA Noma H.; J-SUPPORT 2001 Study group. Optimizing smartphone psychotherapy for depressive symptoms in patients with cancer: multiphase optimization strategy using a decentralized multicenter randomized clinical trial (J-SUPPORT 2001 Study). Psychiatry Clin Neurosci. 2024;78:353–61. 38468404 10.1111/pcn.13657 PMC11488626

[36] Lin PH Lin SK Hsu RJ. Spirit-Quieting Traditional Chinese medicine may improve survival in prostate cancer patients with depression. J Clin Med. 2019;8:218. 30744039 10.3390/jcm8020218 PMC6406565

[37] Lv J Zhu L Wu X Yue H Cui X. Study on the correlation between postoperative mental flexibility, negative emotions, and quality of life in patients with thyroid cancer. Gland Surg. 2021;10:2471–6. 34527559 10.21037/gs-21-424 PMC8411093

[38] Javaloyes N Crespo A Redal MC. Psycho-oncological intervention through counseling in patients with differentiated thyroid cancer in treatment with radioiodine (COUNTHY, NCT05054634): A Non-randomized Controlled Study. Front Psychol. 2022;13:767093. 35282223 10.3389/fpsyg.2022.767093 PMC8914112

[39] Chen Y Ju P Xia Q. Potential role of pain catastrophic thinking in comorbidity patients of depression and chronic pain. Front Psychiatry. 2022;13:839173. 35898637 10.3389/fpsyt.2022.839173 PMC9309267

[40] An Y Liu Z Wang S. Effect of Chaihu plus Longgu Muli decoction plus five-element music therapy in the treatment of cancer-related depression. Support Care Cancer. 2022;30:7955–62. 35737144 10.1007/s00520-022-07172-6

[41] Jung MS Visovatti M Kim M Cha K Dlamini N Cui X. Cognitive impairment in women newly diagnosed with thyroid cancer before treatment. Support Care Cancer. 2022;30:8959–67. 35922683 10.1007/s00520-022-07299-6

[42] Noto B Asmus I Schäfers M Görlich D Riemann B. Predictors of anxiety and depression in differentiated thyroid cancer survivors: results of a cross-sectional study. Thyroid. 2022;32:1077–85. 35734910 10.1089/thy.2022.0067

[43] Pandi-Perumal SR Monti JM Burman D. Clarifying the role of sleep in depression: a narrative review. Psychiatry Res. 2020;291:113239. 32593854 10.1016/j.psychres.2020.113239

[44] Daut RA Fonken LK. Circadian regulation of depression: a role for serotonin. Front Neuroendocrinol. 2019;54:100746. 31002895 10.1016/j.yfrne.2019.04.003 PMC9826732

[45] Yan Z Lai Z Lin J. Anticancer properties of traditional Chinese medicine. Comb Chem High Throughput Screen. 2017;20:423–9. 28093974 10.2174/1386207320666170116141818

[46] Jiang Y Liu LS Shen LP. Traditional Chinese medicine treatment as maintenance therapy in advanced non-small-cell lung cancer: a randomized controlled trial. Complement Ther Med. 2016;24:55–62. 26860802 10.1016/j.ctim.2015.12.006

[47] Hughes DT Reyes-Gastelum D Kovatch KJ Hamilton AS Ward KC Haymart MR. Energy level and fatigue after surgery for thyroid cancer: a population-based study of patient-reported outcomes. Surgery. 2020;167:102–9. 31582311 10.1016/j.surg.2019.04.068 PMC6904434

[48] Xu JD Eniwar Z Kayisa Wu EW Wang DM WufuEr H. Study on Chinese medicine syndrome elements characteristics of lung cancer associated depression. New Chin Med. 2017;49:56–9.

[49] Bortolato B Hyphantis TN Valpione S. Depression in cancer: the many biobehavioral pathways driving tumor progression. Cancer Treat Rev. 2017;52:58–70. 27894012 10.1016/j.ctrv.2016.11.004

[50] McFarland DC Riba M Grassi L. Clinical implications of cancer related inflammation and depression: a critical review. Clin Pract Epidemiol Ment Health. 2021;17:287–94. 35444703 10.2174/1745017902117010287 PMC8985467

[51] Lanser L Kink P Egger EM. Inflammation-induced tryptophan breakdown is related with anemia, fatigue, and depression in cancer. Front Immunol. 2020;11:249. 32153576 10.3389/fimmu.2020.00249 PMC7047328

[52] Szałach LP Lisowska KA Cubała WJ. The Influence of Antidepressants on the Immune System. Arch Immunol Ther Exp (Warsz). 2019;67:143–51. 31032529 10.1007/s00005-019-00543-8 PMC6509093

